# Unlabeled lysophosphatidic acid receptor binding in free solution as determined by a compensated interferometric reader

**DOI:** 10.1194/jlr.D120000880

**Published:** 2020-06-08

**Authors:** Manisha Ray, Kazufumi Nagai, Yasuyuki Kihara, Amanda Kussrow, Michael N. Kammer, Aaron Frantz, Darryl J. Bornhop, Jerold Chun

**Affiliations:** * Degenerative Disease Program, Sanford Burnham Prebys Medical Discovery Institute, La Jolla, CA 92037; † Department of Chemistry and Vanderbilt Institute for Chemical Biology, Vanderbilt University, Nashville, TN 37235; § Biomedical Sciences Graduate Program, University of California San Diego, La Jolla, CA 92037

**Keywords:** receptor binding assay, G protein-coupled receptor, lysophospholipids, molecular interaction, interferometry, free-solution assay-compensated interferometric reader, lipid signaling

## Abstract

Native interactions between lysophospholipids (LPs) and their cognate LP receptors are difficult to measure because of lipophilicity and/or the adhesive properties of lipids, which contribute to high levels of nonspecific binding in cell membrane preparations. Here, we report development of a free-solution assay (FSA) where label-free LPs bind to their cognate G protein-coupled receptors (GPCRs), combined with a recently reported compensated interferometric reader (CIR) to quantify native binding interactions between receptors and ligands. As a test case, the binding parameters between lysophosphatidic acid (LPA) receptor 1 (LPA_1_; one of six cognate LPA GPCRs) and LPA were determined. FSA-CIR detected specific binding through the simultaneous real-time comparison of bound versus unbound species by measuring the change in the solution dipole moment produced by binding-induced conformational and/or hydration changes. FSA-CIR identified *K_D_* values for chemically distinct LPA species binding to human LPA_1_ and required only a few nanograms of protein: 1-oleoyl (18:1; *K_D_* = 2.08 ± 1.32 nM), 1-linoleoyl (18:2; *K_D_* = 2.83 ± 1.64 nM), 1-arachidonoyl (20:4; *K_D_* = 2.59 ± 0.481 nM), and 1-palmitoyl (16:0; *K_D_* = 1.69 ± 0.1 nM) LPA. These *K_D_* values compared favorably to those obtained using the previous generation back-scattering interferometry system, a chip-based technique with low-throughput and temperature sensitivity. In conclusion, FSA-CIR offers a new increased-throughput approach to assess quantitatively label-free lipid ligand-receptor binding, including nonactivating antagonist binding, under near-native conditions.

G protein-coupled receptors (GPCRs) represent a large super-family of membrane-bound signal transducing receptors that are activated by the binding of small molecules. Lysophospholipid (LP) receptors are a subset of GPCRs that mediate the actions of LP signaling lipids and have myriad biological roles throughout the body (1–3). LP receptors include five sphingosine-1-phosphate receptors that are already the target of three US Food and Drug Administration-approved medicines (fingolimod, siponimod, and ozanimod) (4–9) and six lysophosphatidic acid (LPA) receptors for which therapies are under clinical development (10). LPs were among the first bioactive signaling lipids identified (1, 2) and consist of a hydrophilic phosphate head group, a chiral -OH group, and a hydrophobic acyl chain of different lengths and degrees of saturation (11).

The six cognate LPA receptors (LPA_1–6_) activate a range of heterotrimeric G proteins (11); all six receptors have been knocked out in mice revealing diverse biological effects (2, 12–16); and the crystal structures were determined for two LPA receptors (17–19). Despite these advances, it remains difficult to determine the native binding of unlabeled LPs to their cognate receptors in free solution. There are high levels of nonspecific signal produced by partitioning of labeled lipid ligands within cell membranes that enable normal GPCR function. Moreover, receptor binding studies usually employ highly overexpressed and/or modified receptors (e.g., tagged with EGFP), in addition to labeled ligands, which can affect results in unpredictable ways (20). Available biophysical techniques (21–23) like surface plasmon resonance (24, 25), fluorescence resonance energy transfer (26), fluorescence polarization (27), fluorescence cross-correlation spectroscopy (28), and radioligand binding (RLB) (29) all require immobilization and/or ligand labeling, which can affect *K_D_* values as a result of chemical perturbations, such as those from fluorescent dye molecules or structural inflexibility produced by molecular tethers and immobilization.

Interferometric interaction assays have received significant interest over the past two decades to measure the affinity of molecular binding under more native conditions (i.e., in free solution and without labeling) (30–36). Free-solution assays (FSAs) allow for measurement of inherent solution-phase properties such as the conformational or hydration changes produced by binding (31–33). These changes can be detected by the newly developed compensated interferometric reader (CIR) (36, 37). The combination of FSA-CIR should allow for the determination of binding parameters including the dissociation constant (*K_D_*) between various lipid chemical forms and their known and unknown cognate receptors under label-free conditions.

We recently reported LPA-specific binding to LPA_1_ using a predecessor technology, back-scattering interferometry (BSI), which had low throughput (six samples with five replicates; ∼3 h) and variability produced by temperature (35). To overcome these challenges, a new CIR (36) was developed by the Bornhop laboratory at Vanderbilt University (36), which enabled simultaneous measurement of sample and reference-pairs using the same probe beam, thus nullifying sensitivity to temperature fluctuations. The use of a capillary cell for smooth uninterrupted sample introduction and detection enhanced the signal-to-noise ratio and increased throughput compared with the BSI platform.

Here, we report a novel free-solution label-free assay using CIR that produces a 12-fold higher throughput (12 samples with 5 replicates; ∼30 min). FSA-CIR was used to determine LPA-LPA_1_ *K_D_*s for multiple LPA forms with differing acyl chain length and saturation, representing a proof-of-concept for the broader use of FSA-CIR to interrogate LP and other lipid ligand-receptor molecular interactions including orthosteric, allosteric, and antagonist binding.

## MATERIALS AND METHODS

### LPA handling and stock preparation

Various chemical forms of LPA were assayed: 1-oleoyl-LPA (18:1), 1-palmitoyl-LPA (16:1), 1-arachidonoyl-LPA (20:4), 1-linoleoyl-LPA (18:2), and 1-oleoyl-lysophosphatidylcholine (18:1 LPC) (Avanti Polar Lipids Inc.). Saturated or mono-unsaturated samples (16:0, 18:1 LPAs, and 18:1 LPC) were completely dissolved in ethanol:water (1:1 v/v) by sonicating for 3–5 min, aliquoted in glass vials layered with N_2_, and stored under N_2_ atmosphere at −20°C for several uses (up to 9 months). Unstable and unsaturated LPA samples (18:2 and 20:4; received in CHCl_3_) were desiccated and then reconstituted in fresh ethanol:water (1:1 v/v) for immediate use in binding assays. Redissolving desiccated LPAs in aqueous BSA solutions for storage purposes was eliminated because it resulted in 95–97% loss of LPA during reconstitution (38). Stored or reconstituted LPAs in ethanol:water solution show a monodispersed distribution of LPA as measured by dynamic light scattering (DLS). Saturated LPAs are relatively stable under atmosphere, whereas unsaturated ones are highly unstable, extremely hygroscopic, and, therefore, cannot be stored for subsequent use in this assay.

### Preparation of cell lines

Stable B103 cell lines expressing LPA_1_ were developed, cultured, and used for receptor-containing nanovesicle preparation, as previously described (35). Briefly, a polyclonal B103 rat neuroblastoma stable cell line expressing human LPA_1_ with an HA epitope tagged N terminus (HA-LPA_1_-B103) was established by antibiotic selection and cell sorting (35). Microsomal fractions were prepared from HA-LPA_1_-B103 cells and controls (vector transfected cells; Vec-B103) by starving the cells for 16 h in DMEM high glucose containing 0.5% BSA (Gemini Bio Products); the cells were washed with ice-cold PBS, collected by scraping, and stored at −80°C for vesicle preparation.

### Nanovesicle preparation from HA-LPA_1_-B103 and Vec-B103 cells

**Fig. 1.**
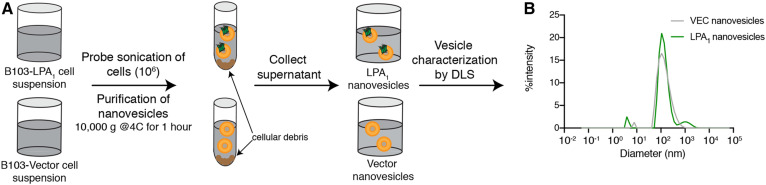
Sample workflow used to prepare and characterize LPA_1_-containing and vector nanovesicles. A rat neural cell line, B103, was used to produce LPA_1_-containing vesicles by heterologous expression of a human LPA_1_ cDNA that was stably expressed. Vector transfected B103 cells were used as a control. A: B103-LPA_1_ and B103-vector transfected cell suspensions were probe sonicated (Qsonica Q125 sonicator; ∼30–40% amplitude; pulse: 5 s on, 1 s off for 90 s), and the resulting nanovesicles were isolated by centrifugation. The nanovesicle- containing supernatant was characterized using the Bradford assay for protein concentration. B: DLS was used to determine vesicle size distributions. Vesicles of diameters ∼100–150 nm were utilized.

HA-LPA_1_-B103 or Vec-B103 cells were probe-sonicated to generate nanovesicles (39) for analysis (**Fig. 1A**). Briefly, HA-LPA_1_-B103 or Vec-B103 cell pellets (∼6–7 × 10^6^ cells) were resuspended in 1 ml of ice-cold PBS containing cOmplete™ protease inhibitor mixture (Roche) and transferred to a glass dram vial. Cell suspensions in an ice bath were then probe sonicated (Qsonica Q125 sonicator, 30–40% amplitude with an intense pulse sound; pulse: 5 s on, 1 s off, for 90 s) and the resulting solutions were centrifuged at 4°C for 1 h at 10,000 *g*. The supernatant containing nanovesicles with HA-LPA_1_ or vector was collected and stored at 4°C until use later that day. The expression of HA-LPA_1_ was confirmed by Western blot (35) with Vec-B103 cells serving as a negative control. Vesicles were characterized using DLS (Dynapro Nanostar, Wyatt Technologies) and total protein concentration was measured by Bradford assay using fatty acid-free BSA as a standard.

### FSA preparation

**Fig. 2.**
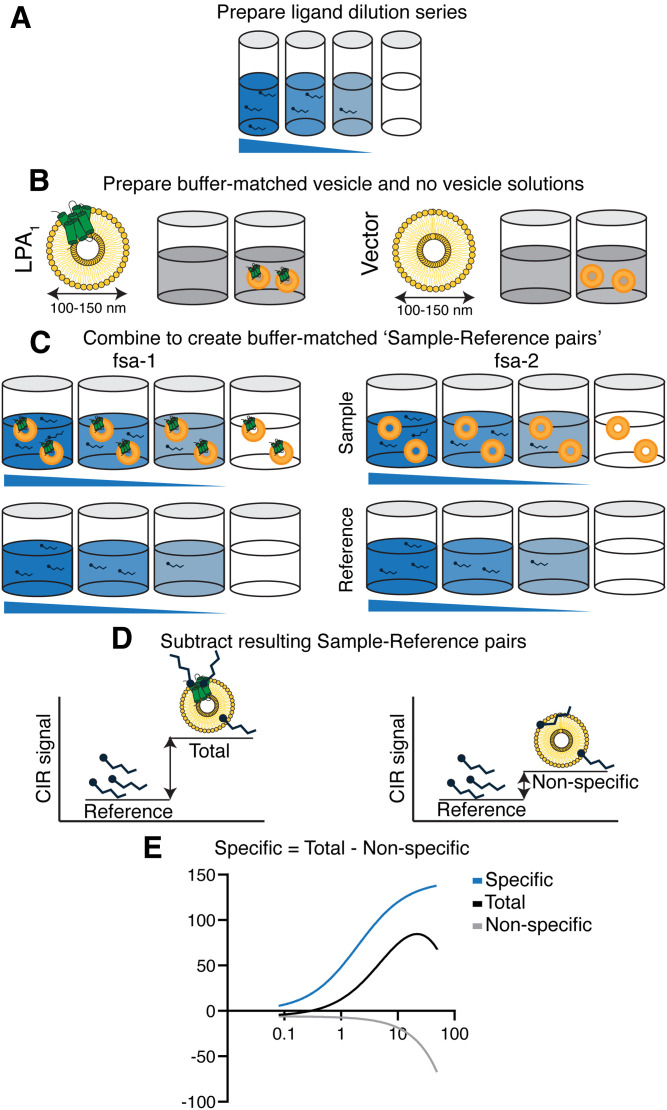
Cell membrane vesicle-based FSA protocol. A: An LPA dilution series was prepared in 0.01% fatty acid-free BSA/0.002% ethanol (six to seven dilutions were prepared for the binding assay). B: Buffer-matched sample-reference pairs were prepared with LPA_1_/no vesicle and vector/no vesicle solutions. C: LPA dilution series were mixed with LPA_1_-containing and vector nanovesicles (test samples) and with the paired buffer-matched no vesicles solution (reference samples) in *fsa-1* and *fsa-2* and were equilibrated for 1 h. D: Sample-reference pairs were processed in the CIR (Fig. 3) with increasing concentrations of LPA and a fixed concentration of total protein (LPA_1_/vector; 25 μg/ml). One binding curve was generated for each sample-reference pair: the vector-sample measures nonspecific signal and the LPA_1_ sample measures total binding signal. E: The specific binding signal (blue) was calculated by subtracting the nonspecific binding signal from the total binding signal. *K_D_* for LPA to LPA_1_ was calculated by plotting the specific binding signal against LPA concentrations.

The FSA preparation was modified from a tissue-based assay protocol (33). Nanovesicle solutions and their buffer-matched vesicle devoid solutions were prepared independently and combined with the LPA dilution series to create index-matched sample-reference pairs (**Fig. 2**).

#### LPA ligand solution preparation.

In blood or plasma, 30–40% of LPA circulates bound to the carrier protein albumin (Fig. 2A). Therefore, freshly prepared fatty acid-free BSA was used in the final binding assay preparation for in vivo compatibility. LPAs have poor solubility, low critical micelle concentration (∼300 μM) and bind to Eppendorf tube walls when prepared in aqueous buffers (40), resulting in concentration variations of the analyte and error in the measurement. LPA bound to fatty acid-free BSA in solution can result in aggregation (diameter ranges from 10 to 10,000 nm) when stored at −20°C, even after reducing the particle size by sonication. Therefore, LPAs were assessed in freshly prepared fatty acid-free BSA solution. A stock solution of LPA in ethanol:water (5 mM) was redissolved in 0.1% fresh fatty acid-free BSA (w/v) solution to prepare 200 nM of intermediate stock containing 0.01% fatty acid-free BSA in 0.002% ethanol/PBS (v/v). The 0.002% ethanol in 0.01% BSA/PBS solution was kept constant across all ligand dilutions to ensure that free solution measurements were index matched.

#### LPA_1_ or vector- and buffer-matched reference solution preparation.

LPA_1_-containing or vector control nanovesicles in solution were made using cOmplete™ protease inhibitor solution in PBS, diluted with 1× PBS (pH 7.4) to a working concentration of 50 μg/ml (Fig. 2B). Buffer-matched no-vesicle solutions were prepared as reference solutions.

### Binding assay preparation

A serial dilution series (100, 20, 4, 0.8, 0.16, 0.032, 0.0064, and 0 nM) of lipid ligands was prepared from a 200 nM of LPA solution by diluting with 0.002% ethanol/0.01% BSA/PBS (Fig. 2A). A “zero” concentration consisted of 0.002% ethanol/0.01% BSA/PBS. Each concentration of the diluted ligand was combined with an equal volume of the 50 μg/ml LPA_1_-containing or vector control nanovesicle solutions (Fig. 2B) to produce binding and nonbinding test samples with buffer-matched no-vesicle reference solutions (Fig. 2C) with a final buffer composition of 0.001% ethanol/water/0.005% BSA in PBS. The final protein concentration was 25 μg/ml and the final ligand concentration ranged from 0 to 50 nM. The mixtures were allowed to reach equilibrium for 1 h at room temperature prior to analysis by CIR.

### The CIR

**Fig. 3.**
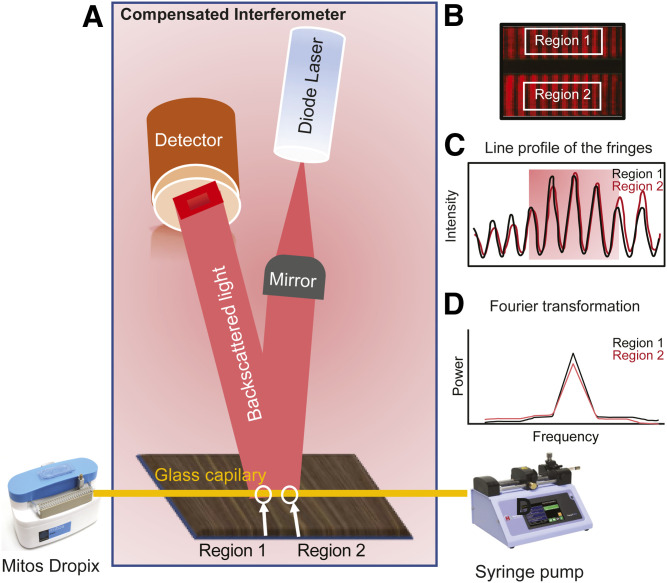
CIR. A: CIR consists of a diode laser, a microfluidic channel (a glass capillary), a fringe detector, an automated droplet generator for sample introduction (Mitos Dropix), and a syringe pump. The Mitos Dropix introduces sample droplet trains into the glass capillary while the syringe pump maintains a constant sample flow through the capillary. Sample and reference pairs flow through regions 1 and 2 where they are simultaneously interrogated by the diode laser. Resultant images of the fringe patterns and their phase shifts under binding/nonbinding conditions (B) are converted to a line profile (C) where selected fringes are fast Fourier transformed for analyses (D).

The simple and cost-effective experimental arrangement of the CIR has been described elsewhere (41, 42) and consists of the compensated interferometer, a droplet generator (Mitox Dropix; Dolomite Microfluidics), and a syringe pump (Harvard Apparatus) (**Fig. 3**). This next generation BSI is a droplet-based technology that allows for simultaneous interrogation of sample and reference in continuous droplet trains separated by thermally and chemically stable oil (Fluorinert FC-40, Sigma-Aldrich).

The interferometer consists of a diode laser, a beam directing optic (one 1/2 mirror), a microfluidic channel (a glass capillary), and a CCD camera (Fig. 3). The auto sample introducer was programed with built-in software to introduce droplet trains of sample-reference pairs into a glass capillary. As demonstrated recently (37), droplet trains of sample-reference pairs were produced by a Dropix sample hook that guides the capillary tubing up and down between sample-reference pairs contained in a bottomless reservoir made of polyether ether ketone materials mounted on a second fluid reservoir (#3200354, Dolomite Microfluidics) containing the Fluorinert™ FC-40 oil (Sigma-Aldrich). The syringe pump pulls fluid from both reservoirs to maintain a constant flow of the droplet train through the capillary while maintaining a constant pressure without perturbation by any other sources. Simultaneous sample-reference interrogation (from region 1 and 2; Fig. 3A, B) was measured by direct probing with an expanded beam profile emanating from the laser diode. The assays were measured sequentially, starting with the reference sample. Briefly, the capillary was filled with rinse buffer (0.005% BSA in 0.001% ethanol/PBS) and the syringe pump flow rate was set to 20 μl/min for 8–10 min to achieve a stable flow. The assay was run by introducing 1 μl of sample-reference pairs (five replicates) followed by two rinses of 2 μl, each separated by a 40 nL droplet of oil. This process was repeated for all concentrations. Prior to analysis of other LPA forms, the glass capillary was completely cleaned with 1 ml of a 1:1 (v/v) mixture of CHCl_3_:methanol and dried manually with a syringe vacuum to eliminate lipid carryover.

The resulting backscattered interference fringes were detected by the CCD array using a detection window of 200 pixels long (1,100 μm) along a glass capillary with an inner diameter of 250 μm, yielding an optical probe volume of 54 nL. The positional shift of the fringes (equivalent to molecular binding) was quantified using a fast Fourier-transform algorithm in a customized Labview™ program.

### Statistical analyses

Each receptor-ligand interaction (isotherm) was repeated at least three times on different days with freshly prepared FSA and each had five to seven replicates. The total versus nonspecific binding CIR signal, as plotted on the y-axis, and different ligand concentrations on the x-axis were fitted using GraphPad Prism™.

Total=specific+nonspecific

Nonspecific=NS×X+Background

$$\mathrm{Specific}=\mathrm{Bmax}\times X/\left(X+K_{D}\right)$$

## RESULTS

### Measurement of monodisperse nanovesicle size distributions

LPA_1_ and control nanovesicles were prepared by probe sonication of microsomal fractions from HA-LPA_1_-B103 and vector-B103 cells (Fig. 1A) to produce nanovesicles with a size distribution of 100–150 nm (as measured by DLS) (Fig. 1B). Monodisperse solutions of LPA_1_ and vector nanovesicles with intense single and overlapping DLS peaks were essential to avoid rapid vesicle fusion and aggregation, as well as possible index mismatch of control solutions. Nanovesicles were used fresh to provide predictable and consistent results: 4°C storage resulted in aggregation and −80°C storage resulted in both aggregation and ice crystal formation.

### FSA

Two sample-reference-pair solutions were used to determine specific binding: *fsa-1* (total binding) and *fsa-2* (nonspecific binding) (Fig. 2). The *fsa-1* sample-reference pair consisted of LPA_1_-vesicle (test sample) and buffer-matched (reference sample) solutions with increasing concentrations of LPA ligand (Fig. 2C). The *fsa-2* sample-reference pair was identical, except that it contained vector control nanovesicles rather than LPA_1_ nanovesicles. The total protein concentration of LPA_1_ or vector-nanovesicles was fixed at 25 μg/ml. The difference in interferometric signal between the sample-reference pair in *fsa-1* provided a quantitative measure of the total binding of LPA ligands to LPA_1_, whereas *fsa-2* provided nonspecific binding of LPA ligands to vector nanovesicles (Fig. 2D, E). Precise preparation of buffer-matched sample-reference pairs and the subsequent subtraction eliminated background signal created by the complex matrix of LPA_1_. Thus, when measured in the CIR, *fsa-1* versus *fsa-2* allowed determination of specific LPA-LPA_1_ *K_D_* values (**Table 1**).

**TABLE 1.** Binding constants (*K_D_*) for different LPA species

Membrane Bound Receptor	Ligands LPA/LPC	*K_D_* ± SEM	Previously Reported *K_D_* Values	Previously Reported EC_50_ Values
LPA_1_	18:1 LPA	2.08 ± 1.32 nM	*K_D_* = 0.87 ± 0.37 nM (from BSI)	200 nM
			*K_D_* = 68.9 nM (from RLB)	
	18:2 LPA	2.83 nM ± 1.64	None reported	200 nM
	20:4 LPA	2.59 nM ± 0.481	None reported	200 nM
	16:0 LPA	1.69 nM ± 0.1	None reported	400 nM
	18:1 LPC	∼0 nM	None reported	None reported

**Fig. 4.**
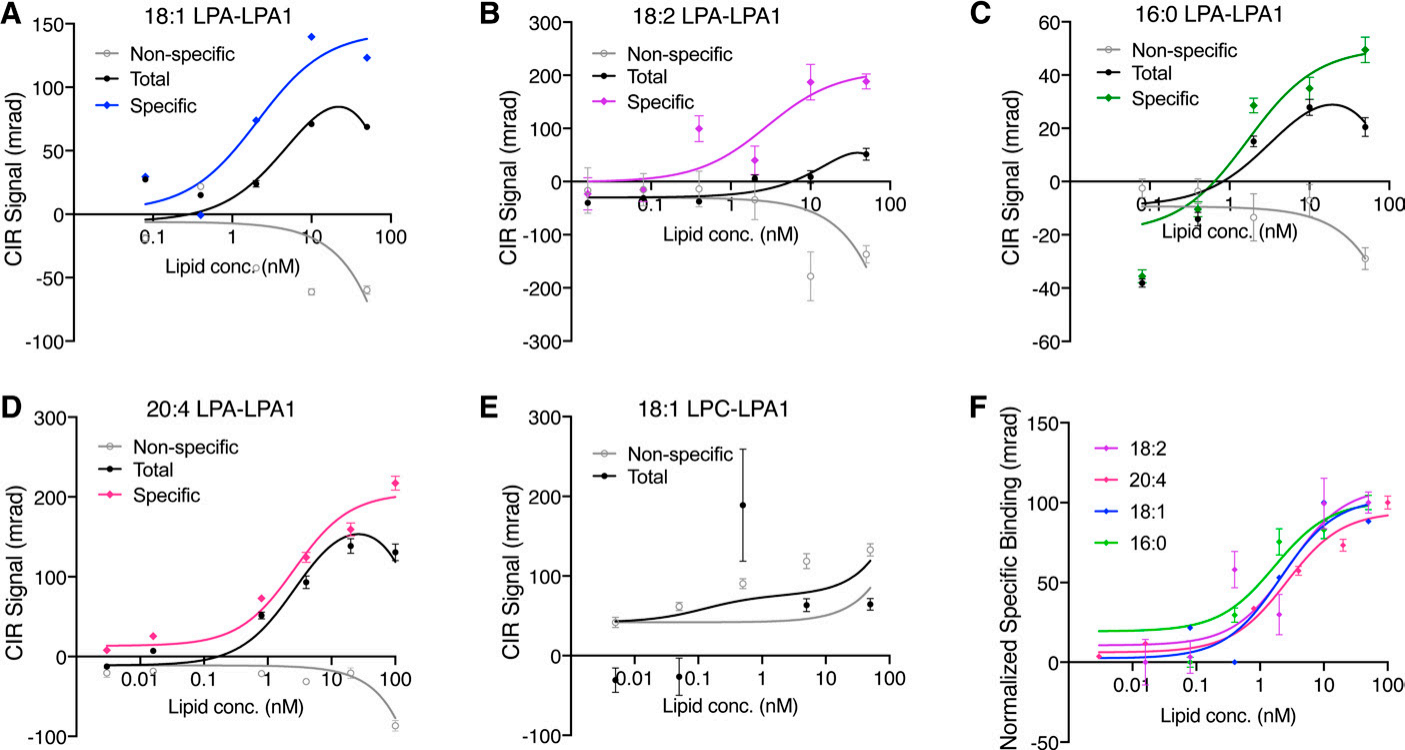
CIR determination of specific binding of LPA ligands 18:1, 18:2, 16:0, and 20:4 to LPA_1_ compared with LPC. CIR signals versus ligand concentration were plotted. A–E: Representative plots of changes in RI (milliradians) produced by binding as revealed by CIR for 18:1 (A), 18:2 (B), 16:0 (C), 20:4 (D) LPA, and 18:1 LPC (E) (negative control). Nonspecific (gray), total (black), and calculated specific (colored) binding are shown. F: Normalized specific binding signal for all LPA ligands overlapped (see Table 1 for *K_D_* values). Each graph shows an average of three independent binding isotherms (experimental replicates), each with five to seven measurements (technical replicates).

Binding constants were determined from specific binding data from the plots (Fig. 4) compared with reported BSI (35), RLB (29), and EC_50_ (46) assessments.

### LPA-specific binding to LPA_1_ in cell membrane nanovesicles identified by FSA-CIR

Five LPA ligands that differed in acyl chain length and saturation were assayed to quantify their binding affinity to a cognate receptor, LPA_1_, as compared with a control LP, LPC (**Fig. 4**). In the CIR, an expanded diode laser beam produces “elongated” fringes resulting from illumination of the droplet train filled capillary. Elongated fringe patterns differ between sample and reference pairs, which translated into RI differences that also changed in proportion to the ligand concentration. Fringe-shift measurements from ligands interacting with LPA_1_ produced the total binding signal (*fsa-1*; Fig. 4A–E, black lines) that showed successively positive RI changes that increased with lipid concentration; subtraction of minor nonspecific RI changes (*fsa-2*; Fig. 4A–E, gray lines) enabled calculation of specific signals (Fig. 4A–E; colored lines) and *K_D_* values were calculated (Table 1; Fig. 4F).

All LPA forms exhibited *K_D_* values in the low nanomolar range [1-oleoyl (18:1) (*K_D_* = 2.08 nM ± 1.32)], 1-linoleoyl (18:2) (*K_D_* = 2.83 nM ± 1.64), 1-arachidonoyl (20:4) (*K_D_* = 2.59 nM ± 0.481), and 1-palmitoyl (16:0) (*K_D_* = 1.69 nM ± 0.1); Table 1] regardless of the acyl chain length or saturation. This is consistent with the documented selectivity of the LPA_1_ binding pocket for the phosphate headgroup rather than the acyl chain (17). No specific signals were observed for total versus nonspecific binding of 18:1 LPC. The specific low nanomolar (2–3 nM) *K_D_* values of LPA_1_-LPA binding demonstrate both the sensitivity and specificity of FSA-CIR, thus supporting its utility in detecting lipid receptor-ligand binding under label-free conditions.

## DISCUSSION

Molecular interaction studies with lipids represent a challenge because of the physical-chemical nature of lipids including ligand solubility, membrane intercalation, loss to surfaces, and stability. Classical receptor-lipid binding assays using radiolabeled ligands are difficult because of the high levels of nonspecific binding within membranes, ligand degradation, and the requirement for receptors to be properly folded within a cell membrane lipid bilayer. Here, we report FSA-CIR that measures such interactions using label-free signaling LPAs and a cognate GPCR (using LPA_1_) in nanovesicles, freely floating in solution. Individual measurement of total and nonspecific binding reduces the background signal produced by assay conditions where GPCRs are present in a complex milieu of other lipids, proteins, and biological fluids. Nanovesicle-based receptor binding FSAs in combination with CIR should be generalizable to measure many other signaling lipids that interact with cell-surface receptors known to regulate myriad cellular and physiological processes (2, 6, 10, 11).

FSA-CIR studies identified a requirement for several key variables: uniform size of nanovesicle, buffer-matched control solutions, fresh nanovesicle preparations, and precise lipid handling. Control of these variables enabled FSA-CIR to achieve substantial improvements over other methods including the previous generation of BSI. Techniques that utilize target and/or ligand immobilization [surface plasmon resonance, BLI (43, 44)] and/or labeling [fluorescence resonance energy transfer, fluorescence polarization, RLB (45)] can alter the binding characteristics of the ligands, receptors, or both, which can obfuscate native binding characteristics. Thus, FSA-CIR better approximates a native binding environment. By comparison, the previous generation BSI assay had limitations related to difficulty of use, sample preparation and delivery, throughput, and temperature sensitivity. FSA-CIR employs semi-automated sample delivery and simultaneous interrogation of sample and reference (29) to reduce instrument noise produced by operator skill level, laser instability, and temperature fluctuations.

FSA-CIR provided comparable detection of *K_D_* values with its predecessor BSI (Table 1) (35). FSA-CIR-measured *K_D_* values ranged from 0.87 to 2.59 nM for all forms of LPA. These *K_D_* values show a 35- to 40-fold higher affinity than previous assessments by RLB (29) that reported *K_D_* values of 68.9 nM for 18:1 LPA-LPA_1_ binding and similar values for other LPA receptors (LPA_2_ *K_D_* = 63.7 nM, LPA_4_ *K_D_* = 99.6 nM, and LPA_5_ *K_D_* = 88.6 nM). The higher nanomolar *K_D_* values (weaker affinity) detected by RLB likely reflect technical and procedural artifacts such as the rapid off-rate caused by several washing steps that may result in high nonspecific binding. This comparison demonstrates the utility of our FSA-CIR approach as a highly sensitive and reliable binding assay. To our knowledge, these data are the first determination of *K_D_* values for other native forms of LPA (16:0,18:2, and 20:4). Our results indicate no specificity of LPA_1_ toward saturated or unsaturated LPA forms, which is comparable to previously reported EC_50_ values from a Ca^+2^ response assay that showed similar potency for all LPA forms to active LPA_1_ and LPA_2_ (Table 1) (46). Other reports identified ligand specificity for other LPA receptors (18, 29, 46–49) and these distinct LPA ligand-receptor interactions remain to be assessed in future FSA-CIR studies.

Importantly, FSA-CIR was able to achieve this sensitivity and specificity with only nanograms (picomoles) of receptor protein. If we assume 100% binding and no free LPA molecules at the 100 nM LPA concentration, only 1.35 ng of total protein (containing ~3.2 × 10^9^ LPA_1_ molecules) are needed to achieve a saturated binding signal. Similarly, at the minimum quantifiable binding signal (500 pM of LPA), 1.6 × 10^7^ (27 attomoles) LPA-LPA_1_ complexes are present. Combined, our assay required 21 μg of total protein to assess all replicates and LPA concentrations, illustrating the small amounts of lipid ligand-receptor complex required to observe a binding signal, and the versatility of this FSA-CIR system.

Altogether, FSA-CIR provided comparable detection to BSI while allowing for ∼12-fold increased throughput. Previously difficult to measure lipid ligand-receptor interactions (50) can now be approached with comparative ease under more native conditions that do not require radioactivity or labeling of ligands or receptors. Notably, the in vivo presence of bivalent cations (e.g., Ca^2+^ and Mg^2+^) will alter the availability and physiology of LPA ligands and, therefore, will likely impact receptor binding affinities (51). Assessment of LPA-LPA_1_ binding under improved physiological conditions is imperative for future drug discovery efforts. These features raise the possibility of examining future samples from primary cells and even tissues naturally or engineered to be devoid of a single target receptor, as well as allowing interrogation of binding interactions that occur in complex matrices like human fluids and tissues. FSA-CIR should thus be useful in identifying and validating a range of lipid ligand-receptor interactions, including those with clinical potential.
